# Transcriptome and hormone Analyses reveal that melatonin promotes adventitious rooting in shaded cucumber hypocotyls

**DOI:** 10.3389/fpls.2022.1059482

**Published:** 2022-11-28

**Authors:** Yuping Wang, Hailiang Zhao, Xiaohui Hu, Yi Zhang, Zicun Zhang, Lu Zhang, Lixia Li, Leiping Hou, Meilan Li

**Affiliations:** ^1^ College of Horticulture, Shanxi Agricultural University, Taigu, Shanxi, China; ^2^ Experimental Teaching Center, Shanxi Agricultural University, Taigu, Shanxi, China; ^3^ College of Horticulture, Northwest A&F University, Yangling, China

**Keywords:** melatonin, cucumber, hypocotyl shade, adventitious roots, hormones, transcriptome

## Abstract

Melatonin, a multi-regulatory molecule, stimulates root generation and regulates many aspects of plant growth and developmental processes. To gain insight into the effects of melatonin on adventitious root (AR) formation, we use cucumber seedings subjected to one of three treatments: EW (hypocotyl exposed and irrigated with water), SW (hypocotyl shaded and irrigated with water) and SM (hypocotyl shaded and irrigated with 100 µM melatonin). Under shaded conditions, melatonin induced significant AR formation in the hypocotyl. To explore the mechanism of this melatonin-induced AR formation, we used transcriptome analysis to identify 1296 significant differentially expressed genes (DEGs). Comparing SM with SW, a total of 774 genes were upregulated and 522 genes were downregulated. The DEGs were classified among different metabolic pathways, especially those connected with the synthesis of secondary metabolites, with hormone signal transduction and with plant-pathogen interactions. Analyses indicate exogenous melatonin increased contents of endogenous auxin, jasmonic acid, salicylic acid, cytokinin and abscisic acid levels during AR formation. This study indicates melatonin promotes AR formation in cucumber seedings by regulating the expressions of genes related to hormone synthesis, signaling and cell wall formation, as well as by increasing the contents of auxin, cytokinin, jasmonic acid, salicylic acid and abscisic acid. This research elucidates the molecular mechanisms of melatonin’s role in promoting AR formation in the hypocotyl of cucumber seedings under shaded conditions.

## Introduction

Cucumber plants have poor ability to take up water and minerals because of their weak root regeneration and shallow roots system, which seriously reduces yield. The hypocotyl of cucumber seedlings has the ability to generate AR (Qi et al., 2019; Qi et al., 2020), therefore measures that stimulate adventitious root development are crucial for promoting plant growth and development and thus for increasing crop yield and so making better use of scarce agricultural land.

Adventitious roots (ARs) can emerge from a hypocotyl, a stem, a leaf or some other plant organ, with emergence regulated by environmental signals and/or by hormones (Sukumar et al., 2013). Many hormones have been shown to play important roles in the induction of AR, among these auxin is the main one that promotes AR initiation (Li et al., 2009; Da Costa et al., 2013; Pacurar et al., 2014).It is reported that auxin synthesis is necessary for AR formation, with the number of ARs increasing with auxin concentration (Boerjan and W., 1995; Ahkami et al., 2013). Conversely, reduced auxin transport or reduced auxin signaling decreases AR development (Vidoz et al., 2010). In addition to auxin, cytokinin (CK), jasmonic acid (JA), salicylic acid (SA) and ethylene are also involved in regulating AR development, and they interact with one another. Studies have shown that CK inhibits AR formation (Hutchison et al., 2006; Mao et al., 2019), over-expression of cytokinin oxidase (*CKX*) increases AR formation in transgenic tobacco (Werner et al., 2001). It is reported that SA works synergistically with auxin to induce AR development in cucumber (Dong et al., 2020) and Arabidopsis (Pasternak et al., 2019). Moreover, ethylene is another important hormone that interacts with auxin during root development; with ethylene inducing AR formation by regulating auxin transport (Vidoz et al., 2010). Moreover, the crosstalk between ethylene and JA signaling has been shown to be critical for AR formation in Arabidopsis (Fattorini et al., 2018).

Melatonin is a natural molecule that exists widely in plants (Pelagio-Flores et al., 2012), which is structurally related to indole-3-acetic acid (IAA). Initially, the structural similarity between melatonin and IAA prompted researchers to investigate its function in greater depth (Hernández-Ruiz et al., 2004). In plants, melatonin may act as a growth regulator or new hormone (Arnao and Hernández-Ruiz, 2014; Arnao and Hernandez-Ruiz, 2019). A growing number of studies have reported the possible physiological roles of melatonin and their mechanisms. They have shown that melatonin can perform a number of roles, such as protecting the photosynthetic system and related subcellular structures (Zhao et al., 2016; Altaf et al., 2021), promoting seed germination (Zhang et al., 2014; Hwang and Back, 2020) and providing effective defenses against both biotic and abiotic stresses (Zhang et al., 2017; Li et al., 2018). Moreover, with auxin-like activity, melatonin is able to stimulate root initiation and induce root growth. In recent years, a large number of studies have confirmed that applications of exogenous melatonin can promote root development, especially the development of lateral roots and AR. For example, exogenous melatonin can promote the emergence of lateral roots and AR in lupin (Arnao and Hernández‐Ruiz, 2007) and Arabidopsis (Pelagio-Flores et al., 2012), and can promote AR formation in tomato (Wen et al., 2016) and apple (Mao et al., 2020).

Previous studies have shown that melatonin is an important modulator of gene expression related to hormone production, as well as of the metabolism of auxin, CK, JA, SA and ethylene and other endogenous hormones (Arnao and Hernandez-Ruiz, 2018). For example, melatonin regulates root development by increasing the level of auxin and inducing the expression of genes related to auxin signal transduction, transport and synthesis (Chen et al., 2009; Wen et al., 2016; Liang et al., 2017; Mao et al., 2020). However, applications of exogenous melatonin at high concentration, inhibit root growth by inhibiting auxin transport and synthesis, and is accompanied by a decrease in endogenous IAA (Wang et al., 2016). Under copper stress, melatonin promotes root development in melon by inhibiting the expression of JA synthesis-related genes (Hu et al., 2020). In Arabidopsis, melatonin promotes the expressions of IAA and ethylene synthesis-related genes, whereas it inhibits the expressions of brassinosteroid, JA and CK synthesis-related genes, thereby integrating hormone signals to further mediate the expressions of zinc finger proteins and calmodulin-like proteins, so as to promote lateral roots development (Yang et al., 2021a). Moreover, melatonin also upregulates SA-related genes, leading to an increase in SA, while melatonin synthesis enzyme serotonin N-acetyltransferase mutants exhibited lowered levels of SA (Lee et al., 2015). Though these studies demonstrate a link between melatonin and hormones, the relationship between melatonin and these hormones in the formation of AR in cucumber seedlings is unknown.

To better understand how melatonin induces AR formation in the hypocotyls of cucumber seedlings under shaded conditions, we first analyze the effects of melatonin on AR formation and hormone content in cucumber hypocotyls. Then, combined with transcriptomics analysis, we explore the mechanism of AR formation induced by exogenous melatonin under shaded hypocotyl conditions. This research aimed to explore the effects of melatonin on AR development in cucumber seedlings, and to provide a basis for revealing the mechanism of melatonin in regulating AR formation in cucumber seedlings.

## Materials and methods

### Plant materials

Seeds of cucumber (*Cucumis sativus* L.) cultivar ‘Xinyan 4’ were soaked in 55°C water for 12 h to trigger germination, then placed in a growth chamber under conditions of photoperiod (day/night, 12/12 h), light intensity (300 µmolm-2 s-1), temperature (28/18°C) and relative humidity (75%). When the second true leaf was fully expanded, seedlings were divided randomly into three groups. Each group was subjected to one of three treatments: EW (hypocotyl exposed to air, irrigated with water), SW (hypocotyl shaded with sand, irrigated with water) and SM (hypocotyl shaded with sand, irrigated with 100 µM melatonin) and then cultivated for three days. The hypocotyls were then isolated and analyzed for hormone content and subjected to transcriptome analysis. Each treatment was repeated three times, with 180 seedlings per treatment.

### Morphological observation of adventitious roots

Three days after treatment, adventitious root development in the hypocotyl was determined under a stereomicroscope. Ten seedlings were collected 12 days after treatment and the numbers of AR in the hypocotyl were counted.

### Determination of hormone content

Hormone determination of the freeze-dried hypocotyl was carried out with three biological replicates by UPLC-MS/MS (LCMS-6500 system, Sciex) according to the method described previously (Flokova et al., 2014; Cui et al., 2015; Guo et al., 2021). Briefly, hormone was extracted by crushing the frozen sample in a mixer mill (MM 400, Retsch) for 1.0 min at 30 Hz under freezing conditions. Next, 50 mg of the frozen powder was extracted with a methanol: water: formic acid mix (15:4:1, v:v:v) and injected into a QTRAP 6500+ LC-MS/MS system (Sciex, USA). Multiquant 3.0.3 software (Sciex) was used to analyze the data.

### RNA extraction and library construction

The RNA was extracted from the hypocotyl tissue (three replicates per treatment). The RNA-Seq was commissioned to Metware Biotechnology Co. Ltd. (Wuhan, China). Sequencing libraries were constructed using Illumina’s NEBNext UltraTM RNA Library Prep Kit (NEB, USA). Firstly, magnetic beads with oligo (dT) were used to enrich mRNA, and the mRNA were broken into fragment by adding fragmentation buffer. Then added M-MuLV Reverse Transcriptase, random hexamer primer, RNase H and DNA Polymerase I to synthesize double-stranded cDNA. Library was constructed after purification and amplification. Sequencing was carried out on the Illumina HiSeq platform.

### Transcriptomic data analysis

Clean data were produced by removing reads of low-quality, containing poly-N and adapter sequences from raw reads. HISAT2 tools was used to map the clean data to the cucumber reference genome sequence (http://cucurbitgenomics.org/organism/20). The gene expression levels were calculated as fragments per kb of transcript per million reads (FPKM) values. We used the DESeq2 package to conduct differentially expressed genes (DEGs) analyses. Genes with |log_2_FC|≥1 and padj ≤ 0.05 were considered to be DEGs. KEGG pathway analysis and GO analysis were carried out for all DEGs.

### Quantitative real-time PCR validation

The qRT-PCR was carried out on a Step One Plus Real-Time Fluorescent Quantitative PCR system (ABI, USA) using a SYBR Premix EX Taq kit (Takara, Japan). Quantification was evaluated using the 2^−(ΔΔCt)^ method. Specific primers of 16 genes randomly selected from the DEGs were listed in **Supplementary Table S2**.

## Results

### Effects of melatonin on adventitious root development

**Figure 1:**
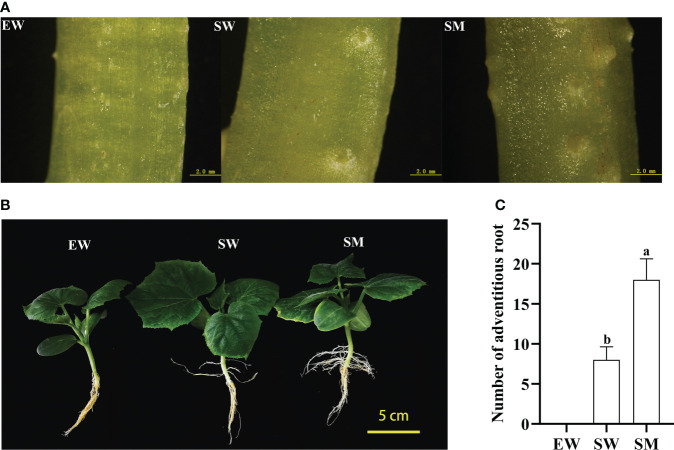
Effects of melatonin on adventitious root (AR) development of cucumber seedings under hypocotyl shading conditions. **(A)** Observation of AR primordium development with stereoscope after 3 days under EW, SW, and SM treatment (scale bar = 2 mm). **(B)** Observation of AR morphological after 12 days under EW, SW, and SM treatment (scale bar = 5 cm). **(C)** Number of AR after 12 days under EW, SW, and SM treatment. EW (hypocotyl exposed and irrigated with water); SW (hypocotyl shaded and irrigated with water); and SM (hypocotyl shaded and irrigated with 100 µM melatonin). Different letters indicate significant differences according to a Duncan’s multiple range test (P < 0.05).

After three days of treatment, AR primordia on hypocotyls could be seen in the SW and SM treatments, with SM showing more AR primordia than SW (**Figure 1**). The rooting rate in the SM treatments was significantly higher than in the SW treatments after 12 days, while no ARs appeared in the EW treatment (**Figure 1**). The number of AR in the SM treatment was 18, that is 2.25-fold higher than in the SW treatment (**Figure 1**). The results suggest that exogenous melatonin promotes AR development in the shaded hypocotyls of cucumber seedlings.

### Differentially expressed genes induced in hypocotyls by melatonin under shaded conditions

Three cDNA libraries were obtained by sequencing RNA extracted from the EW, SW and SM hypocotyls. Each library consisted of more than 7.1 Gb of clean data. The Q20 percentage was more than 95%, and the error rate was less than 0.04%, indicating high sequencing quality (**Table 1**).

**Table 1:** Summary of sequence data.

Sample	Clean Reads	Clean Base (G)	Error Rate (%)	Q20(%)	GC Content (%)
EW	49380905	7.41	0.04	95.58	43.35
SW	48687753	7.3	0.04	95.55	42.94
SM	47758435	7.16	0.04	95.66	42.26

EW (hypocotyl exposed and irrigated with water), SW (hypocotyl shaded and irrigated with water) and SM (hypocotyl shaded and irrigated with 100µM melatonin).

**Figure 2:**
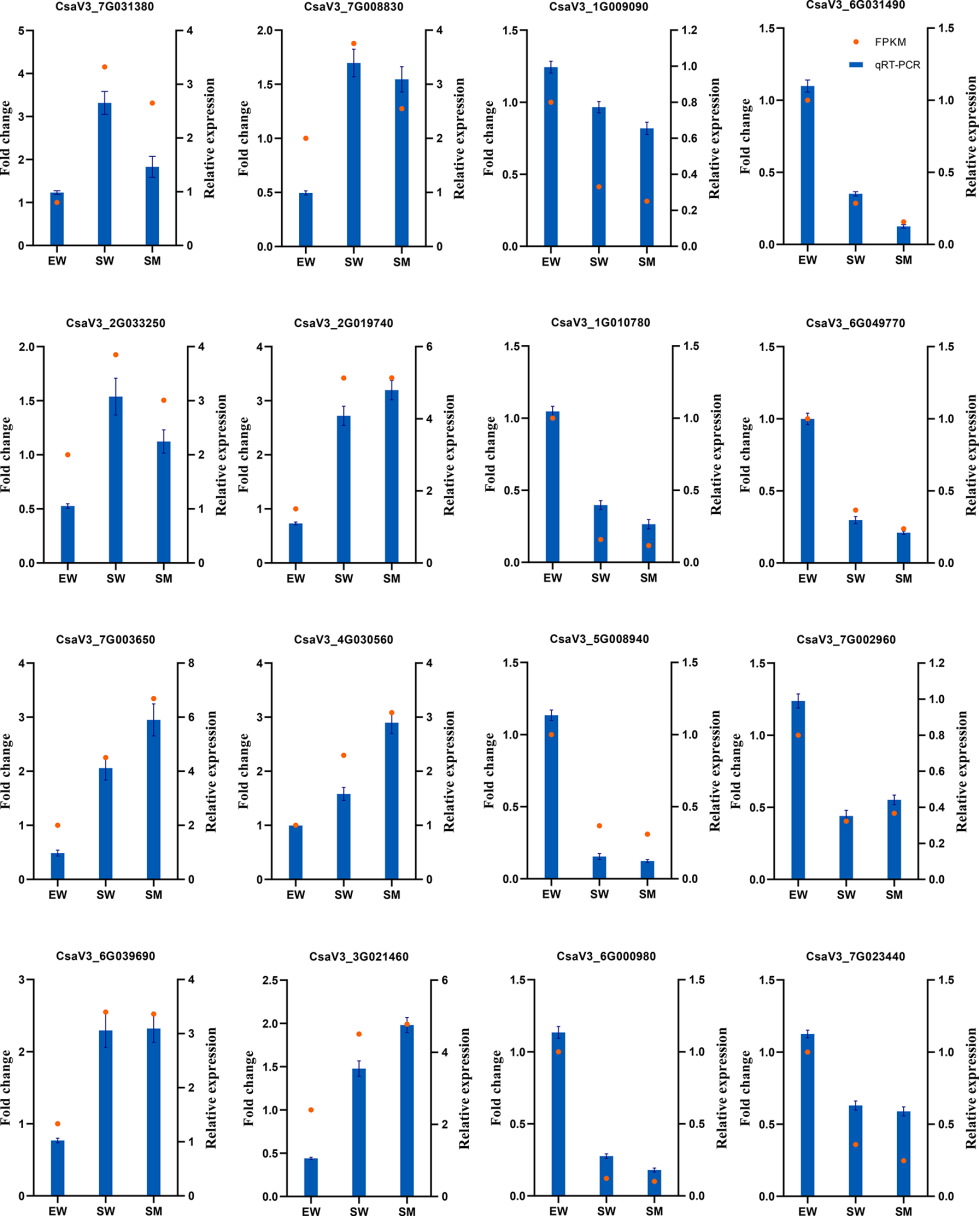
qRT-PCR validation of DEGs detected by RNA-Seq.

To verify the reliability of the RNA-Seq results, 16 DEGs were randomly selected for qRT-PCR verification. The qRT-PCR data were consistent with the RNA-seq data (**Figure 2**), the correlation coefficient is R^2^ = 0.8283 (**Supplementary Figure S1**). These results validate the reliability of the RNA-seq data.

**Figure 3:**
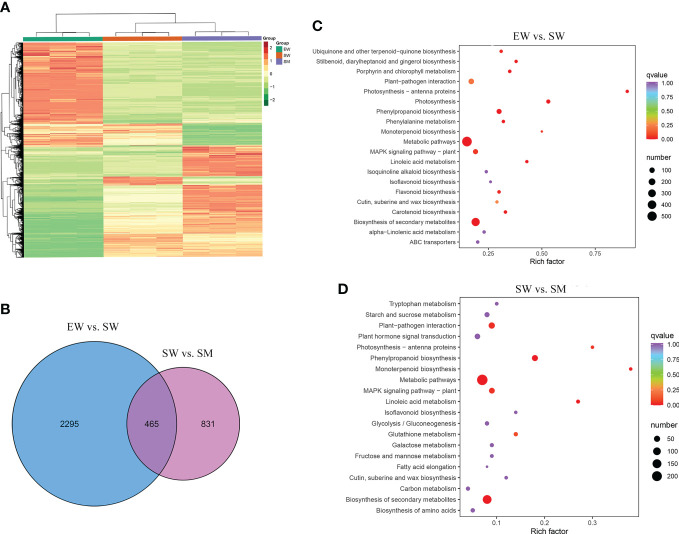
The differentially expressed genes (DEGs) induced by melatonin under hypocotyl shading conditions. **(A)** Hierarchical clustering of DEGs. **(B)** Venn diagram of unique and common DEGs. **(C, D)** KEGG pathways enrichment analysis of DEGs. EW (hypocotyl exposed and irrigated with water), SW (hypocotyl shaded and irrigated with water) and SM (hypocotyl shaded and irrigated with 100 µM melatonin).

To elucidate the molecular regulation mechanism of exogenous melatonin on AR formation in cucumber seedlings, DEGs were analyzed in two comparison groups (EW vs. SW and SW vs. SM). Hierarchical cluster analysis was carried out to observe the overall expression pattern of DEGs, with red and green bands representing high and low genes expression levels, respectively (**Figure 3**). A total of 2760 DEGs were detected in EW and SW, with 1379 genes upregulated and 1381 genes downregulated relative to those in EW. A total of 1296 DEGs were detected in SW and SM, with 774 genes upregulated and 522 genes downregulated relative to those in SW (**Figure 3**). These DEGs were induced by exogenous melatonin and so are important candidate genes for further study.

We carried out a KEGG enrichment analysis of the DEGs to identify the main pathways enriched in the melatonin-induced AR development process. Between EW and SW, the top 20 enriched KEGG pathways were mainly related to biosynthesis of secondary metabolites, metabolic pathways, photosynthesis, MAPK signaling pathways and phenylpropanoid biosynthesis (**Figure 3**). Between SW and SM, the major enriched KEGG pathways being biosynthesis of secondary metabolites, metabolic pathways, plant hormone signal transduction, plant-pathogen interaction and phenylpropanoid biosynthesis (**Figure 3**). We also conducted a GO enrichment analysis of the DEGs to identify the main enriched biological process categories. Between EW and SW, DEGs were significantly enriched in the secondary metabolic process, photosynthesis, response to water deprivation and other biological process categories (**Supplementary Figure S2**). Between SW and SM, DEGs were mainly categorized into the secondary metabolic process, regulation of hormone levels, cell wall biogenesis, phenylpropanoid biosynthetic process, response to fungus and other biological process categories (**Supplementary Figure S2**). From the above, it is clear that DEGs induced by melatonin were significantly enriched in regulation of hormone levels and hormone signal transduction. So, we focused on the effects of melatonin treatment on hormone contents and their related genes.

### Contents of auxin and cytokinin and expression analyses of related genes

**Figure 4:**
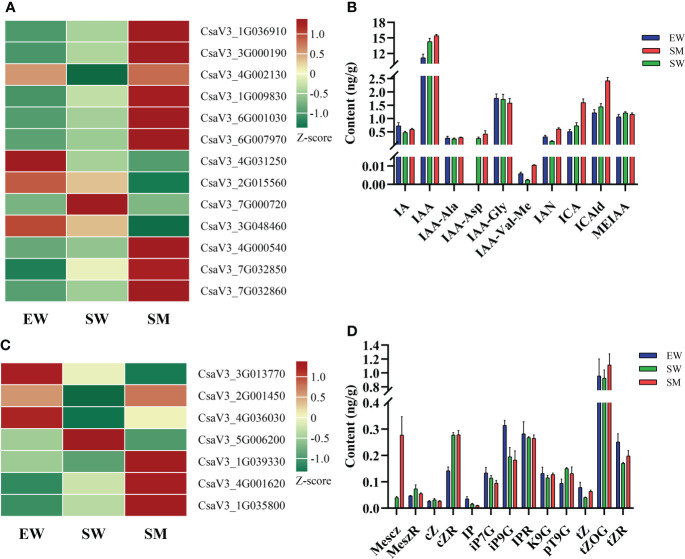
Effects of melatonin on genes and hormones related to auxin and cytokinin in cucumber seedings. **(A, B)** Effects of melatonin on the expression of auxin-related genes and auxin content. **(C, D)** Effects of melatonin on the expression of cytokinin-related genes and cytokinin content. EW (hypocotyl exposed and irrigated with water), SW (hypocotyl shaded and irrigated with water) and SM (hypocotyl shaded and irrigated with 100 µM melatonin).

We found that irrigation with water and melatonin and with shaded hypocotyl the auxin content was significantly affected and also the expressions of the auxin-related genes, involved in auxin synthesis (*TDC, YUCCA* and *TAA*), decomposition (*DAO*), transport (*PIN7*) and signaling (*SAUR*, *AUX/IAA* and *GH3*). The contents of IAA and indole-3-acetyl-L-aspartic acid (IAA-Asp) in SW increased significantly, while indole-3-acetyl-L-valine methyl ester (IAA-Val-Me) and 3-Indoleacetonitrile (IAN) were significantly decreased in SW relative to in EW. Whereas compared with SW, the contents of IAA-Val-Me and IAN increased in SM 3.4-time and 3.1-times, respectively. Moreover, the contents of indole-3-carboxylic acid (ICA) and of indole-3-carboxaldehyde (ICAld) in SM also increased significantly (**Figure 4**). From the transcriptome analysis, we found that the expressions of tryptophan decarboxylase (*TDC*) (CsaV3_1G036910), *PIN7*(CsaV3_3G000190), small auxin-up RNA (*SAUR71*) (CsaV3_1G009830), *SAUR36* (CsaV3_6G001030)*, SAUR21* (CsaV3_7G000720) and 2-oxoglutarate-dependent dioxygenase *(DAO)* (CsaV3_7G032850) were significantly upregulated in SW relative to in EW. Also, the expressions of these genes (except *SAUR21*) in SM were upregulated further relative to those in SW (**Figure 4**). However, compared with that in EW, the expressions of Gretchen Hagen (*GH3.17*) (CsaV3_4G002130), *SAUR32*(CsaV3_4G031250), Flavin-containing monooxygenase (*YUCCA*) (CsaV3_3G017920 and CsaV3_6G012670) and tryptophan aminotransferase (*TAA*) (CsaV3_1G001250 and CsaV3_1G001250) were significantly downregulated in SW. Among these, *GH3.17* was upregulated, while *SAUR32* was downregulated in SM relative to those in SW. *YUCCA* and *TAA* which did not change significantly in SM (**Figure 4**, **Supplementary Table S1**). The SM treatment significantly induced the expressions of *DAO* (CsaV3_4G000540 and CsaV3_7G032860) but inhibited the expression of auxin/indole-3-acetic acid (*AUX/IAA26*) (CsaV3_3G048460) (**Figure 4**, **Supplementary Table S1**).

The content of CK and the expressions of genes related to CK synthesis (*IPT*), conjugation (*UGT76C1*), decomposition (*CKX*) and signal transduction (*AHP*) were also regulated by melatonin under shaded conditions. Compared with that in EW, the contents of 2-Methylthio-cis-zeatin riboside (2MeScZR) and cis-Zeatin riboside (cZR) were significantly increased in SW, while that of N6-isopentenyladenine (IP) was significantly decreased. The level of 2-Methylthio-cis-zeatin (2MeScZ) in SM was increased 5.97-times relative to that in SW (**Figure 4D**). The expressions of cytokinin dehydrogenase (*CKX*) (CsaV3_2G001450 and CsaV3_4G036030) in SW were downregulated relative to those in EW, while in the SM treatment these genes were significantly upregulated. In SW, however, compared with in EW, the expressions of histidine kinase (*AHP6*) (CsaV3_4G001620) and UDP-glycosyltransferase (*UGT76C1*) (CsaV3_1G035800) were upregulated, the expressions of these genes in SM were further upregulated relative to those in SW (**Figure 4C**, **Supplementary Table S1**). Lastly, the expressions of *tRNA-IPT* (CsaV3_3G013770) and *CKX* (CsaV3_5G006200) were significantly inhibited under shaded conditions, while *AHP1* (CsaV3_1G039330) was induced by melatonin (**Figure 4C**, **Supplementary Table S1**).

### Contents of salicylic acids and jasmonic acids and expression analyses ofrelated genes

**Figure 5:**
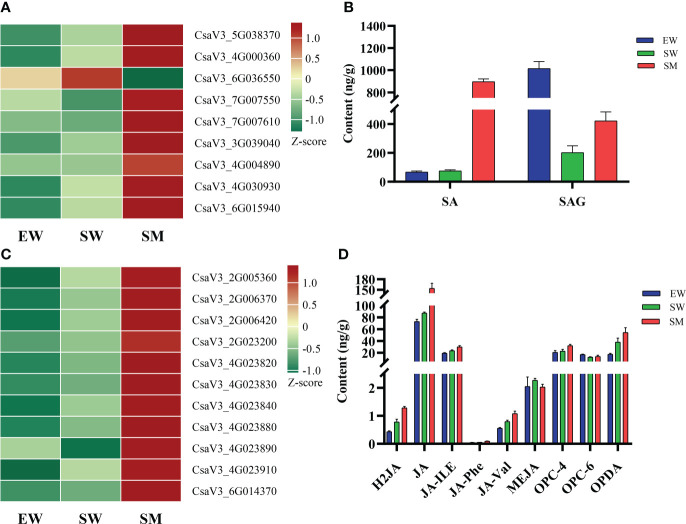
Effects of melatonin on genes and hormones related to salicylic acid and jasmonic acid in cucumber seedings. **(A, B)** Effects of melatonin on the expression of salicylic acid-related genes and salicylic acid content. **(C, D)** Effects of melatonin on the expression of jasmonic acid-related genes and jasmonic acid content. EW (hypocotyl exposed and irrigated with water), SW (hypocotyl shaded and irrigated with water) and SM (hypocotyl shaded and irrigated with 100 µM melatonin).

We found that melatonin induced significant accumulations of salicylic acid 2-O-β-glucoside (SAG) and SA under shaded conditions, with contents of SA increasing by 10.9-times relative to that in SW (**Figure 5B**). However, the content of SAG in SW was significantly decreased, while SA did not change significantly compared with in EW (**Figure 5B**). Melatonin also regulated the expressions of genes related to SA synthesis (*CM*, *PPA-ATs* and *PAL*), conjugation (*UGT74F2*) and signaling (*PR*). Compare with EW, the SW treatment significantly upregulated the expressions of chorismate mutase (*CM*) (CsaV3_5G038370), prephenate aminotransferase (*PPA-ATs*) (CsaV3_4G000360) and UDP-glycosyltransferase (*UGT74F2*) (CsaV3_6G015940), while the expressions of these genes in SM were upregulated further relative to in SW (**Figure 5A**, **Supplementary Table S1**). In addition, the SM treatment significantly upregulated the expressions of *PR* (CsaV3_7G007550 and CsaV3_7G007610) and of transcription factor *TGA* (CsaV3_3G039040, CsaV3_4G004890 and CsaV3_4G030930), while it downregulated the expression of phenylalanine ammonia-lyase (*PAL*) (CsaV3_6G036550) relative to in SW (**Figure 5A**, **Supplementary Table S1**).

Under shaded conditions, melatonin significantly increased the contents of dihydrojasmonic acid (H2JA), N-[(-)-Jasmonoyl]-(L)-valine (JA-Val), jasmonoyl-L-isoleucine (JA-ILE) and cis(+)-12-Oxophytodienoic acid (OPDA). It also significantly promoted accumulations of JA, N-[(-)-Jasmonoyl]-(l)-phenalanine (JA-Phe) and 3-oxo-2-(2-(Z)-Pentenyl) cyclopentane-1-butyric acid (OPC-4) (**Figure 5D**). Irrigation with water and melatonin and with shaded hypocotyl the expressions of genes related to JA synthesis (*LOX*) and signaling (*JAZ* and *MYC2*) was significantly affected. Transcriptome analysis showed that compared with in EW, the expressions of eight lipoxygenase (*LOX*) genes (CsaV3_2G005360, CsaV3_2G006370, CsaV3_2G006420, CsaV3_4G023820, CsaV3_4G023830, CsaV3_4G023840, CsaV3_4G023880 and CsaV3_4G023910) were upregulated in SW and the expressions of these genes were even further upregulated in SM (**Figure 5C**, **Supplementary Table S1**). However, compared with in EW, the SW treatment downregulated the expressions of jasmonate ZIM-domain proteins (*JAZ*) (CsaV3_1G041270) and did not significantly change those in SM (**Supplementary Table S1**). In addition, two *LOX* (CsaV3_2G023200 and CsaV3_4G023890) and one transcription factor *MYC2* (CsaV3_6G014370) were significantly upregulated in SM, relative to in SW (**Figure 5C**).

### Contents of ethylene and abscisic acid and expression analyses of related genes

**Figure 6:**
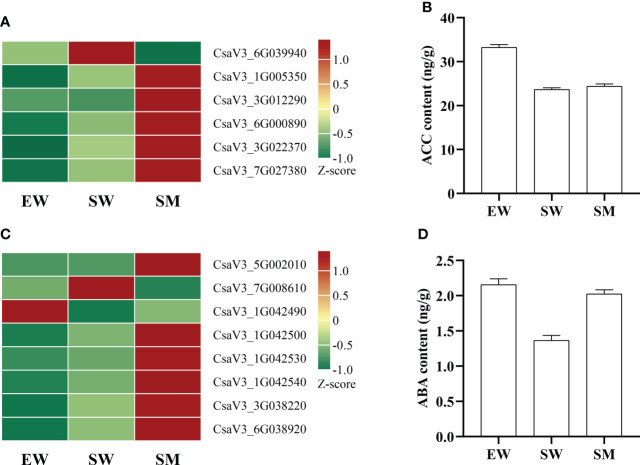
Effects of melatonin on genes and hormones related to ethylene and abscisic acid in cucumber seedings. **(A, B)** Effects of melatonin on the expression of ethylene-related genes and ethylene content. **(C, D)** Effects of melatonin on the expression of abscisic acid-related genes and abscisic acid content. EW (hypocotyl exposed and irrigated with water), SW (hypocotyl shaded and irrigated with water) and SM (hypocotyl shaded and irrigated with 100 µM melatonin).

The content of 1-Aminocyclopropanecarboxylic acid (ACC) in SW decreased significantly relative to in EW, but it did not change significantly in SM (**Figure 6B**). ACC synthase (ACS) and ACC oxidase (ACO) are key enzymes in ethylene synthesis. We found that, compared with in EW, the expressions of ethylene-responsive transcription factor (*ERF1*) (CsaV3_3G022370 and CsaV3_7G027380) in SW were upregulated. The expressions of these genes were even further upregulated in SM (**Figure 6A**). However, the expressions of *ACO* (CsaV3_3G012240 and CsaV3_3G023650) in SW were significantly downregulated, and these genes did not change significantly in SM (**Supplementary Table S1**). However, compared with in SW, the expressions of *ACO* (CsaV3_1G005350 and CsaV3_3G012290) and *ACS* (CsaV3_6G000890) were significantly upregulated in SM, while S-adenosylmethionine synthase (*SAMs*) (CsaV3_6G039940) was significantly downregulated (**Figure 6A**).

The content of ABA was significantly decreased by shading but significantly increased by melatonin under shading (**Figure 6D**). The expressions of genes related to ABA synthesis (*ABA2*), metabolism (*ABA 8′-hydroxylase, β-glucosidase*) and signal transduction (*CDPK*) were also regulated by melatonin. We found that the expressions of β-glucosidase *(β-Glu)* (CsaV3_1G042500, CsaV3_1G042530 and CsaV3_1G042540) and *ABA 8′-hydroxylase* (CsaV3_7G008610) were significantly upregulated by shading, and the expressions of these genes (except of *ABA 8′-hydroxylase*) were further upregulated by melatonin under shaded conditions, while *ABA 8′-hydroxylase* was inhibited by melatonin (**Figure 6C**, **Supplementary Table S1**). However, the expression of *β-Glu* (CsaV3_1G042490) was downregulated by shading, while it was upregulated by melatonin under shaded. Melatonin also significantly induced the expressions of short-chain dehydrogenase (*ABA2*) (CsaV3_5G002010) and calcium dependent protein kinase (*CDPK*) (CsaV3_3G038220 and CsaV3_6G038920) under shaded conditions (**Figure 6C**).

### Expressions of cell wall formation-related genes

We found that in SM, compared with in EW, the expressions of *expansin* (CsaV3_7G001490 and CsaV3_7G001530), nine peroxidase *(POD)* (CsaV3_2G014160, CsaV3_4G023640, CsaV3_4G023650, CsaV3_4G023680, CsaV3_5G012840, CsaV3_6G005630, CsaV3_6G043090, CsaV3_6G043930, CsaV3_7G005720) and cellulose synthase (*CesA*) (CsaV3_1G033150) were upregulated. The SM treatment further upregulated the expressions these genes (except *CesA*) (**Supplementary Figure S3**, **Supplementary Table S1**). The SM treatment upregulated the expressions of *expansin* (CsaV3_7G001510 and CsaV3_7G001520), of ten *POD* (CsaV3_7G030390, CsaV3_7G030400, CsaV3_7G030420,CsaV3_1G030170, CsaV3_2G016300, CsaV3_4G023590, CsaV3_4G023660, CsaV3_4G023670, CsaV3_6G006890 and CsaV3_7G003750) and cellulose synthase-like *(CSL)* (CsaV3_7G011490), and downregulated the expressions of xyloglucan glycosyltransferase (CsaV3_5G037390) and *CesA* (CsaV3_2G009180 and CsaV3_6G007440) significantly (**Supplementary Figure S3**, **Supplementary Table S1**).From these results, it is clear that exogenous melatonin significantly affected the expressions of genes related to cell wall formation under hypocotyl shading conditions.

## Discussion

The formation of AR is affected by water, minerals, wounding and hormones. Many studies have shown that hormones contribute greatly to AR formation (Hutchison et al., 2006; Vidoz et al., 2010; Da Costa et al., 2013; Dong et al., 2020). Melatonin treatments could regulate endogenous hormone contents and their related genes (Arnao and Hernandez-Ruiz, 2018) and so stimulate root generation, giving rise to new lateral and adventitious roots (Arnao and Hernández‐Ruiz, 2007; Pelagio-Flores et al., 2012; Mao et al., 2020). Our results show that exogenous melatonin significantly promotes AR formation in cucumber seedings under hypocotyl shading conditions. We measured hormones levels in the hypocotyls among the three treatments and found that melatonin significantly induced the accumulation of the hormones - auxin, CK, SA, JA and ABA. The GO and KEGG analyses showed that DEGs induced by melatonin were significantly enriched in regulation of hormone levels and plant hormone signal transduction, suggesting that hormones and hormone signal transduction may play important roles in cucumber AR formation.

Melatonin and auxin are tryptophan-derived compounds, previous studies have shown that melatonin may increase auxin contents or exhibit auxin-like activity during root development. For example, melatonin regulates root development in apple (Mao et al., 2020), tomato (Wen et al., 2016), rice (Liang et al., 2017) and Arabidopsis (Yang et al., 2021b) by inducing the expression of genes related to auxin synthesis, transport and signaling, as well as increasing auxin contents. However, other studies have shown that melatonin can regulate root development independently of auxin signaling in Arabidopsis (Pelagio-Flores et al., 2012; Zia et al., 2019). In this study, we found that exogenous melatonin induced AR development in cucumber seedlings may be related to auxin. It has been reported that, transport and accumulation of auxin are necessary for root development, PIN proteins are responsible for auxin transport, *pin1, pin3* and *pin7* mutants show statistically significant reductions in AR formation (Sukumar et al., 2013), while down regulation of the *PIN5* promotes lateral root development (Ren et al., 2019). In this study, it was found that compared with EW, the SW treatment induced the expressions of tryptophan decarboxylase (*TDC*) (CsaV3_1G036910) and *PIN7* (CsaV3_3G000190), whereas it inhibited the expressions of genes related to auxin synthesis, such as *YUCCA* (CsaV3_3G017920 and CsaV3_6G012670) and *TAA*(CsaV3_1G001250) (**Figure 4A**, **Supplementary Table S1**); and decreased the level of IAN, while it increased the levels of IAA and IAA-Asp (**Figure 4B**). Which may be related to the upregulation of the auxin transport gene *PIN*, resulting in local accumulations of IAA. The melatonin (SM) treatment further activated the expression of *TDC* (CsaV3_1G036910) and *PIN7* (CsaV3_3G000190) (**Figure 4A**) under hypocotyl shading conditions. It also increased the contents of IAN significantly (**Figure 4B**), whereas the content of IAA did not increase significantly in SM. This may be related to the upregulation of *DAO* (CsaV3_4G000540 and CsaV3_7G032860), which convert IAA to bound state. It is clear that the increased level of IAA induced by hypocotyl shading is required for the formation of AR (**Figure 1**, **Figure 4B**), while exogenous melatonin significantly promoted the accumulation of IAN under hypocotyl shading conditions (**Figure 4B**), which may be the factor inducing the significant increase in AR. Auxin signaling plays a vital role in the transition of protocambial cells to AR-initiating cells (Chen et al., 2016), auxin induced primary auxin responsive genes include *AUX/IAA*, *SAUR* and *GH3*. In the absence of auxin, Aux/IAAs protein interacts with ARFs and prevents ARFs from binding to the promoter of auxin-reponsive genes. In the presence of auxin, the Aux/IAA protein is degraded through the ubiquitination pathway, then ARFs are released from inhibition and can regulate the expressions of auxin-responsive genes (Weijers and Jürgens, 2004). *SAUR* are highly induced in response to auxin, which regulate various aspects of plant development, such as: root initiation, cell division and cell differentiation. Studies show that SAURs may function as positive regulators of cell expansion, which plays vital roles in IAA-mediated adventitious rooting in petunia cuttings (Yang et al., 2019). *GH3* controls auxin homeostasis in plants, *GH3.3, GH3.5, GH3.6* are required for AR initiation in the Arabidopsis hypocotyl (Gutierrez et al., 2012), *GH3.17* is necessary to control meristem size of the root (Di Mambro et al., 2019). The expression of *SAUR71* (CsaV3_1G009830) and *SAUR36* (CsaV3_6G001030) were upregulated, *GH3.17* (CsaV3_4G002130) was downregulated by hypocotyl shading, and the expressions of these genes were upregulated by melatonin under hypocotyl shading conditions (**Figure 4A**). In addition, melatonin inhibited the expression of *AUX/IAA26* (CsaV3_3G048460) under hypocotyl shading conditions (**Figure 4A**, **Supplementary Table S1**). These results show that hypocotyl shading treatment promoted signal transduction of auxin, and the application of exogenous melatonin under hypocotyl shading conditions further induced auxin signaling, which may promote the formation and elongation of AR. This observation is consistent with the effects of auxin in root induction and elongation (Guan et al., 2019; Qi et al., 2019). From the results, it is clear that the induction effect of melatonin on AR formation in the cucumber seedling hypocotyl may be related to auxin, and there may be interactions between melatonin and auxin. Similar results have been obtained in studies with lupin (Arnao and Hernández‐Ruiz, 2007), tomato (Wen et al., 2016) and apple (Mao et al., 2020).

Cytokinin can regulate cell division, vascular development and root development (Werner et al., 2001; Sakakibara, 2006). It has been reported that, exogenous applications of CK inhibits both lateral (Hutchison et al., 2006; Li et al., 2006) and adventitious root formation (Mahonen et al., 2006) in Arabidopsis. Isopentenyltransferase (IPT) is a key enzyme in CK synthesis, while CK dehydrogenase (CKX) catalyzes the oxidative decomposition of CK (Sakakibara, 2006). Over-expression of IPT can significantly inhibit the development of primary and lateral roots, while over-expressing CKX significantly promotes AR formation in Arabidopsis seedlings (Werner et al., 2001). Transcriptome data analysis found that the expressions of *CKX* (CsaV3_2G001450 and CsaV3_4G036030) were downregulated by hypocotyls shading (**Supplementary Table S1**), whereas the content of IP was decreased (**Figure 4D**), which may be related to the upregulation of *UGT76C1* (CsaV3_1G035800) and glycosylate free-state IP to the bound state. However, melatonin downregulated the expression of *tRNA-IPT* (CsaV3_3G013770) and upregulated the expressions of *CKX* (CsaV3_2G001450 and CsaV3_4G036030) under hypocotyl shading conditions (**Figure 4C**, **Supplementary Table S1**); while the level of 2MeScZ was increased significantly in SM (**Figure 4D**). It has been reported that, 2MeS-CKs (unbound methylthiolated CKs) is less cytotoxic and more resistant to CKX than classical CKs, which may be responsible for continuous tissue proliferation during plant-pathogen interactions (Gibb et al., 2020). In this study, the level of 2MeScZ in SM was significantly increased relative to that in SW, which may be related to the stronger resistance of 2MeScZ to *CKX* and the reduction of decomposition, resulting in local accumulations of 2MeScZ. In addition, KEGG enrichment analysis found the plant-pathogen interaction pathway was significantly enriched in the SW and SM comparison groups (**Figure 3D**). This may be related to pathogenic bacterial infection during AR formation. The accumulation of 2MeScZ in hypocotyls, can mediate plant defenses, promote tissue proliferation around the site of infection and ease deleterious effects on AR development, thereby facilitating the formation of AR. *AHPs* plays a vital role in CK signal transduction, most *AHPs* are positive regulators of CK signal transduction (Hutchison et al., 2006), whereas *AHP6* is a negative regulator of CK signal transduction (Mahonen et al., 2006). In Arabidopsis, reduced AHP function in ahp1,2,3,4,5 mutants resulted in a short primary root but enhanced AR development. Additionally, AHP2, AHP3 and AHP5 are particularly important and AHP1 and AHP4 may play minor roles in these processes (Hutchison et al., 2006). *ahp6* mutants grown on media with increasing concentrations of CK, produce fewer ARs in Arabidopsis (Mahonen et al., 2006). In our study, we found that compared with that in EW, the SW treatment induced the expression of *AHP6* (CsaV3_4G001620), while SM treatment activated *AHP6* expression further (**Figure 4C**, **Supplementary Table S1**). This suggests that melatonin inhibited CK signaling, which may enhance AR development.

Salicylic acid affects seed germination, root elongation and AR development, which is also involved in defense against pathogens. Studies have shown that SA and auxin act synergistically to regulate AR development (Pasternak et al., 2019; Dong et al., 2020). Exogenous SA promoted AR formation in wheat under flooding condition (Koramutla et al., 2022), whereas Arabidopsis *eds5-1* and *eds5-2* mutants lacking SA synthesis produced fewer AR than the wild type. This suggests SA is a positive regulator of AR development (Gutierrez et al., 2012). In Arabidopsis, melatonin upregulated SA-related genes, resulting in an increased level of SA (Lee et al., 2015). In our study, we found that melatonin significantly upregulated the expression of SA synthetase *CM* (CsaV3_5G038370) and *PPA-ATs* (CsaV3_4G000360) (**Figure 5A**, **Supplementary Table S1**), thereby significantly increasing the level of SA in the hypocotyl (**Figure 5B**). Moreover, UDP-glycosyltransferase *UGT74F2* (CsaV3_6G015940) was also upregulated by melatonin, which glycosylated SA to SAG, resulting in a significant increase in SAG in the SM plants. These results indicate that the significant increase in AR induced by exogenous melatonin may be related to increased levels of SA. This is consistent with the effect of SA in cucumber hypocotyl AR induction (Dong et al., 2020). On the other hand, in the process of AR emergence, the young plant is susceptible to affection by soil-borne pathogens. KEGG enrichment analysis found that the plant-pathogen interaction pathway was significantly enriched in the SW and SM comparison groups (**Figure 3D**). This may be related to a pathogenic bacterial infection during AR formation. To ensure plant growth in an unfavorable environment, SA plays a vital role in regulating the growth-defense balance. The expression of pathogenesis-related gene 1(PR1) is considered a marker for activating SA-mediated systemic acquired resistance. It has been reported that, SA inhibited lateral root formation, enhanced adventitious rooting, promoted cell wall lignification and plasmodesmal closure by inducing the expressions of PR1 and PR2, which can directly prevent pathogens from invading roots and so facilitate plants growth under adverse conditions (Bagautdinova et al., 2022). In our study, we found that under hypocotyl shading conditions, exogenous melatonin significantly induced the expressions of *PR1* (CsaV3_7G007550 and CsaV3_7G007610) and of transcription factors *TGA* (CsaV3_3G039040, CsaV3_4G004890 and CsaV3_4G030930) (**Figure 5A**, **Supplementary Table S1**). These can mediate systemic acquired resistance, effectively resisting pathogen infection during AR formation and so facilitating AR development.

It is reported that JA serves as a signal to activate rooting, thereby promoting AR formation in tobacco (Fattorini et al., 2009), pea (Rasmussen et al., 2015), petunia (Lischweski et al., 2015) and Arabidopsis (Fattorini et al., 2018). LOX is a key enzyme in JA synthesis. We found that most LOX genes were upregulated by hypocotyl shading (**Figure 5C**, **Supplementary Table S1**), thereby significantly increasing the content of Jasmonoyl-L-isoleucine (JA-ILE) (**Figure 5D**). Meanwhile, melatonin significantly further upregulated the expressions of these genes under hypocotyl shading conditions (**Figure 5C**, **Supplementary Table S1**), as well as promoting the accumulation of JA-ILE and JA (**Figure 5D**). JAZ proteins are key repressors of JA signaling. MYC2, a bHLH TF, is considered to be a master regulator of the JA signaling (Fernandez-Calvo et al., 2011). In our study, it was found that the expression of *JAZ* (CsaV3_1G041270) was significantly downregulated by hypocotyl shading, and the expression of *MYC2* (CsaV3_6G014370) was significantly upregulated by melatonin under hypocotyl shading conditions (**Figure 5C**, **Supplementary Table S1**). These findings indicate that the formation of ARs induced by hypocotyl shading may be related to the accumulation of JA-ILE and enhanced signaling. Meanwhile, the significant accumulation of JA-ILE and JA, accompany the upregulation of *MYC2* induced by exogenous melatonin may further activate the expression of JA-responsive genes, thereby significantly promoting AR formation, in accordance with the finding that JA acts as a accelerator of AR formation in tea plants (Fan et al., 2021).

Ethylene interacts with auxin, gibberellin and ABA to regulate AR formation in tomato (Vidoz et al., 2010) and rice (Steffens and Sauter, 2006). ACS and ACO are key synthase genes of ethylene, which are upregulated during AR formation in petunia (Lischweski et al., 2015). In Arabidopsis, *ACS* and *ACO* are significantly regulated by melatonin (Yang et al., 2021a). Our Transcriptome data analysis showed that the expressions of *ACO* (CsaV3_3G012240 and CsaV3_3G023650) were downregulated by hypocotyl shading (**Supplementary Table S1**), thereby the level of ACC in SW decreased significantly (**Figure 6B**). However, the melatonin upregulated the expressions of *ACO* (CsaV3_1G005350 and CsaV3_3G012290) and *ACS* (CsaV3_6G000890), while it downregulated the expression of *SAMs* (CsaV3_6G039940) under hypocotyl shading conditions (**Figure 6A**, **Supplementary Table S1**). Also, the ACC content did not change significantly (**Figure 6B**). Many ethylene response factors (*ERFs*) are common mediators of root developmental. It has been found that ERFs are upregulated during AR development in cuttings (Druege et al., 2016). In our study, we found melatonin significantly enhanced the expressions of *ERF1* (CsaV3_3G022370 and CsaV3_7G027380) under hypocotyl shading conditions (**Figure 6A**, **Supplementary Table S1**). The above results show that the significant increase in AR in our cucumber seedlings may well be related to the enhanced expression of ethylene synthesis and response factor genes.

Earlier studies found that ABA can promote AR formation in cucumber (Li et al., 2016) and mung bean seedlings (Li et al., 2014) under stress conditions. ABA 8’-hydroxylase catalyzes the oxidative degradation of ABA, while β-glucosidase (β-Glu) can hydrolyze ABA gluconate into bioactive ABA. Studies on tea plants showed that ABA content was higher in the easy-to-root cultivar, which indicate that ABA is involved in the occurrence of AR (Fan et al., 2021). In our study, the expression of *β-Glu* (CsaV3_1G042500, CsaV3_1G042530 and CsaV3_1G042540) were significantly upregulated by hypocotyl shading and melatonin further upregulated these genes under hypocotyl shading conditions, *while ABA 8′-hydroxylase* was inhibited by melatonin (**Figure 6A**, **Supplementary Table S1**). Thereby the level of ABA in SM increased significantly (**Figure 6D**), which will contribute to the occurrence of AR. Additionally, ABA regulates stomatal transpiration and also root hydrophilicity. One prominent response of roots to ABA sensitivity is their ability to perceive differences in water potential and translate this into growth towards water, which depends on ABA signaling kinase SnRK2.2 activity (Dietrich et al., 2017). Calcium dependent protein kinase (CDPK) is a positive effector of ABA signaling transduction and plays a key role between ABA signal perception and response. In our study, we found that exogenous melatonin significantly induced the expressions of *CDPK* (CsaV3_3G038220 and CsaV3_6G038920) under hypocotyl shading conditions (**Figure 6C**, **Supplementary Table S1**). The upregulation of *CDPK* could phosphorylate downstream target proteins to respond to ABA signals, and so improve the absorptive capacity for water. This too will facilitate the production of AR. These findings indicate that ABA may be involved in melatonin-induced AR development in our cucumber seedlings.

Cellulase-like synthases (CSL) are the main load-bearing components of cell walls. AtCSLD3 is very important for the synthesis of primary cell wall in the root hair region and plays vital roles in root hair growth. In *AtCSLD3* deficient plants, the growth of root hairs is affected (Xuan et al., 2001). Peroxidase (POD) induces cell wall loosening and cell elongation by regulating the elongation and cross-linking of cell wall components (Passardi et al., 2004). From our analyses, we found that melatonin significantly induced *POD* expression under hypocotyl shading conditions (**Supplementary Figure S3**, **Supplementary Table S1**). In melon, the expression of POD was also upregulated by melatonin during lateral root development (Hu et al., 2020). The *expansin* can restore the expansion of heat-inactivated cell walls under acidic conditions, which loosen the cell wall, thereby affecting the extensibility of the cell wall (McQueen-Mason and S., 1992). In transgenic tobacco plants over-expressing *TaEXPB23*, the number of lateral roots was significantly increased (Han et al., 2014). In our study, we found exogenous melatonin significantly induced the expression of *expansin* under hypocotyl shading conditions (**Supplementary Figure S3**, **Supplementary Table S1**). The upregulation of *expansin* will lead to the loosening of the cell wall where the new lateral roots appear, which will reduce the mechanical resistance of the stem tissue, thereby aiding the emergence of new ARs. Therefore, exogenous melatonin regulates the formation of cell walls by upregulating the expression of *CSL*, *POD* and *expansin* under hypocotyl shading conditions, thereby significantly inducing AR formation in our cucumber seedlings hypocotyl.

## Conclusion

**Figure 7:**
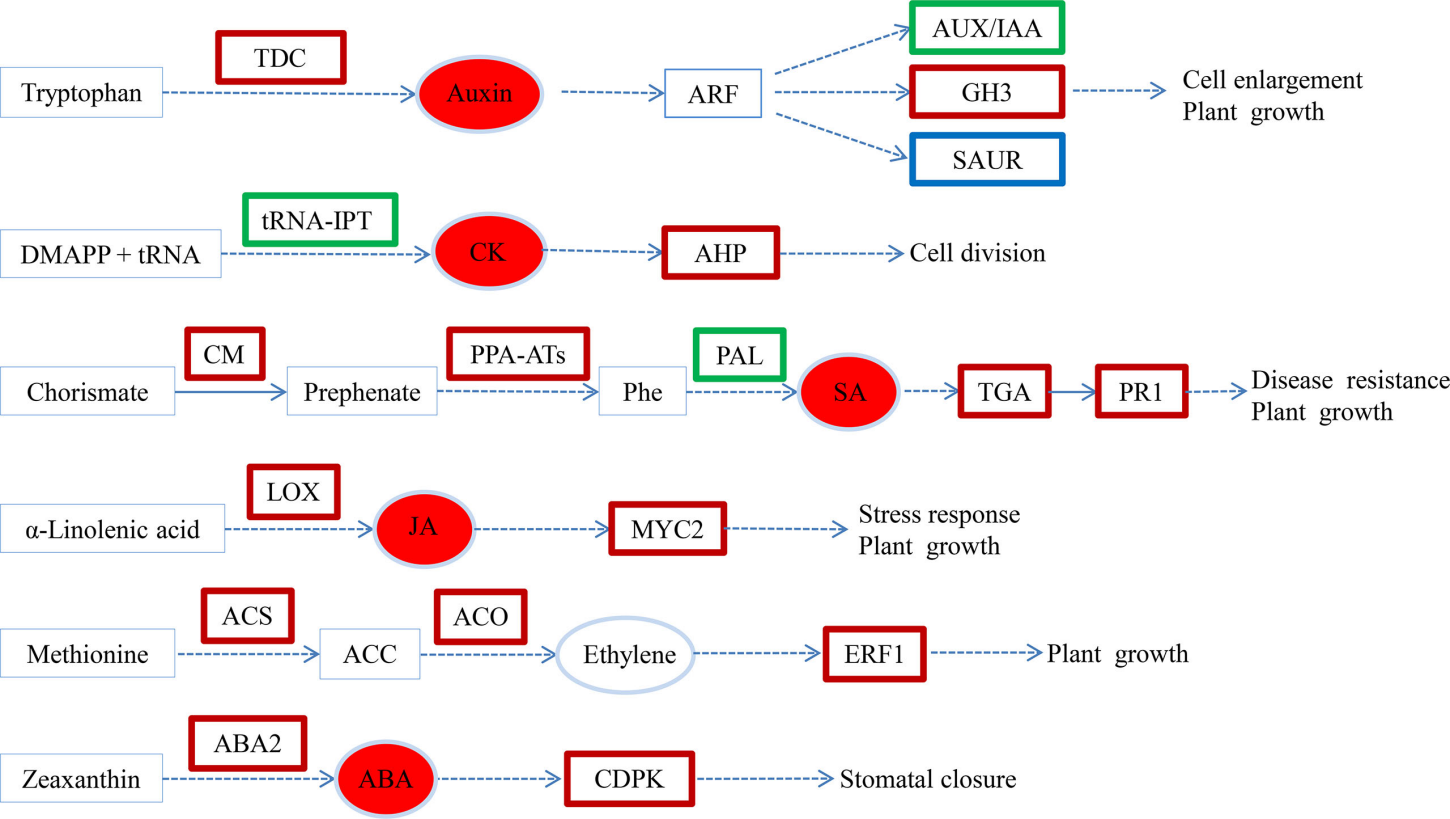
Transcriptional changes in hormone biosynthesis and signaling pathways in SM compared to SW. Upregulated, downregulated and both up- and downregulated genes are boxed in red, green and blue, respectively. The solid arrow indicates a direct step, and the broken arrow indicates an indirect step. SW (hypocotyl shaded and irrigated with water), SM (hypocotyl shaded and irrigated with 100 µM melatonin).

Hypocotyl shading was necessary to induce AR formation, while exogenous melatonin application significantly promoted AR formation in cucumber seedlings under hypocotyl shading conditions. To gain a deeper understanding of the underlying regulatory mechanisms, RNA-seq and hormone analyses were carried out. Our results show that exogenous melatonin induced the expressions of auxin, SA, JA, ethylene and ABA synthesis and signaling related genes. It also inhibited the expressions of CK signaling genes, as well as significantly increasing the auxin, CK, JA, SA and ABA contents (**Figure 7**). In the process of AR formation, melatonin also regulated the expressions of genes related to cell wall formation and induced the loosening of cell walls, thereby facilitating the emergence of AR. Finally, melatonin promoted the formation of a large numbers of AR in the cucumber seedling hypocotyls.

## Data availability statement

The original contributions presented in the study are publicly available. This data can be found here: https://www.ncbi.nlm.nih.gov/bioproject/PRJNA717318. 

## Author contributions

YW, ZZ and LZ carried out experimental work. LL, XH, YZ and LH performed the data analyses. YW wrote the manuscript. HZ and ML designed the experiments and assisted with writing the manuscript. All authors contributed to the article and approved the submitted version.

## Funding

This research was supported by Shanxi Province Key R&D Plan (202102140601013), the National Key R&D Program of China (2019YFD1001900), Shanxi Province Applied basic Research Program (201801D221304), Science and Technology Innovation Fund of Shanxi Agricultural University (2017YJ44, 2018YJ05).

## Conflict of interest

The authors declare that the research was conducted in the absence of any commercial or financial relationships that could be construed as a potential conflict of interest.

## Publisher’s note

All claims expressed in this article are solely those of the authors and do not necessarily represent those of their affiliated organizations, or those of the publisher, the editors and the reviewers. Any product that may be evaluated in this article, or claim that may be made by its manufacturer, is not guaranteed or endorsed by the publisher.

## Supplementary material

Supplementary Figure 1 Relationship between RNA-seq and qRT-PCR expression data (log2 fold change).

Supplementary Figure 2 GO enrichment analysis of DEGs.

Supplementary Figure 3 Effects of melatonin on the expression of cell wall formation-related genes in cucumber seedings.
