# Supplementary figures and images for: Transcriptome and hormone Analyses reveal that melatonin promotes adventitious rooting in shaded cucumber hypocotyls
Source: Front Plant Sci. 2022 Nov 28;13:1059482. doi: 10.3389/fpls.2022.1059482 (PMC9742233; doi:10.3389/fpls.2022.1059482)

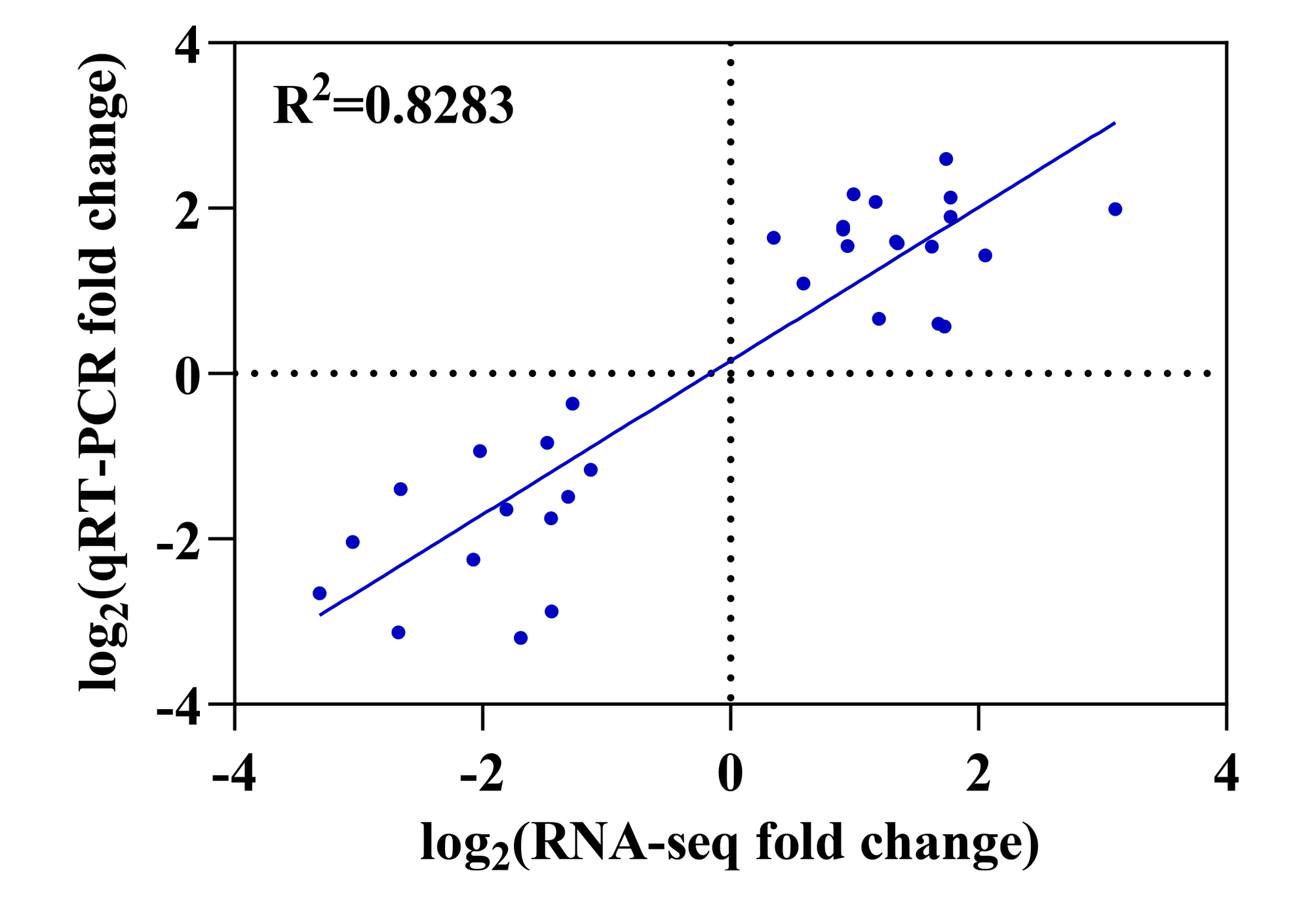

Supplement: Supplementary Figure 1 — Relationship between RNA-seq and qRT-PCR expression data (log2 fold change). [file Image_1.tif]

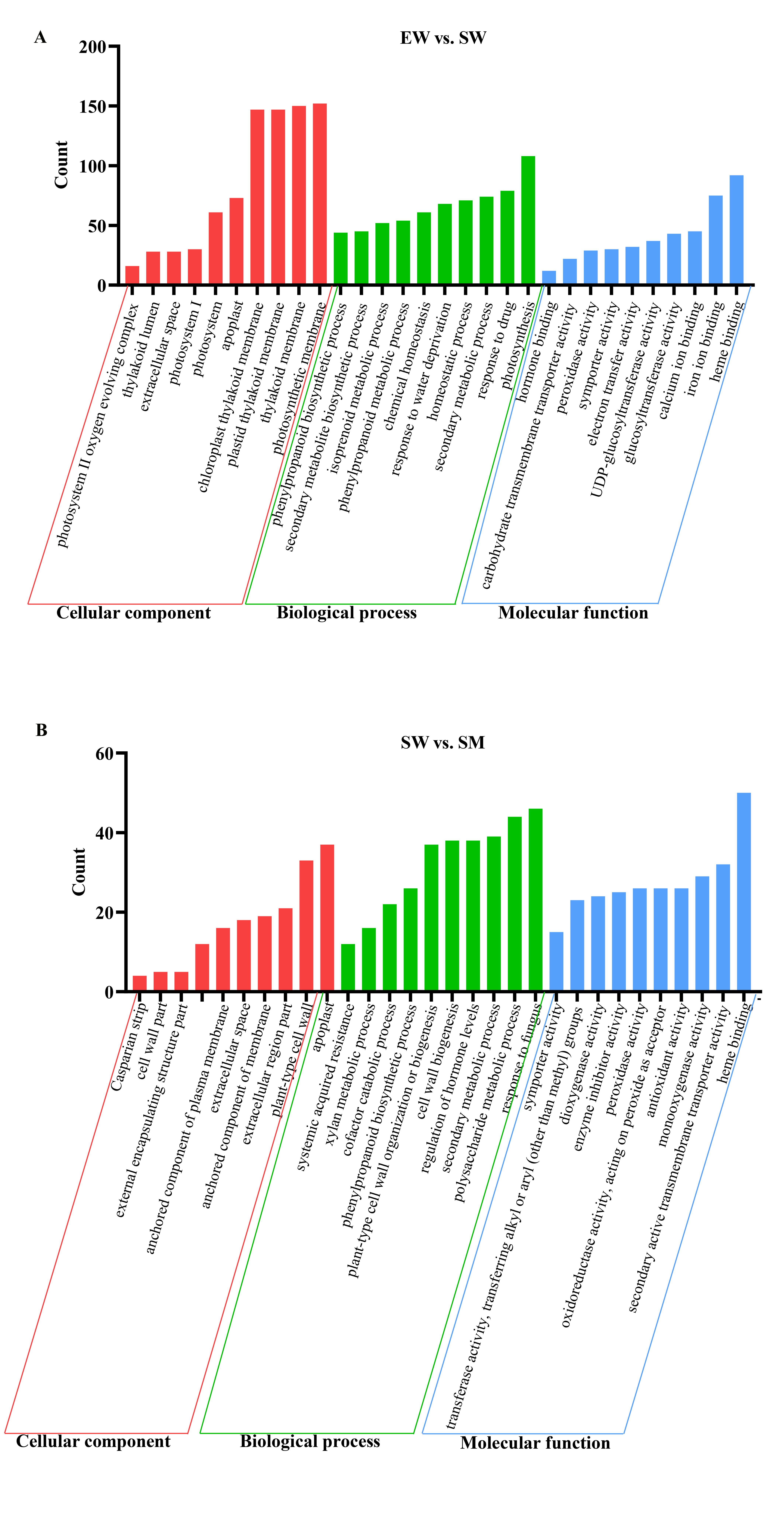

Supplement: Supplementary Figure 2 — GO enrichment analysis of DEGs. [file Image_2.tif]

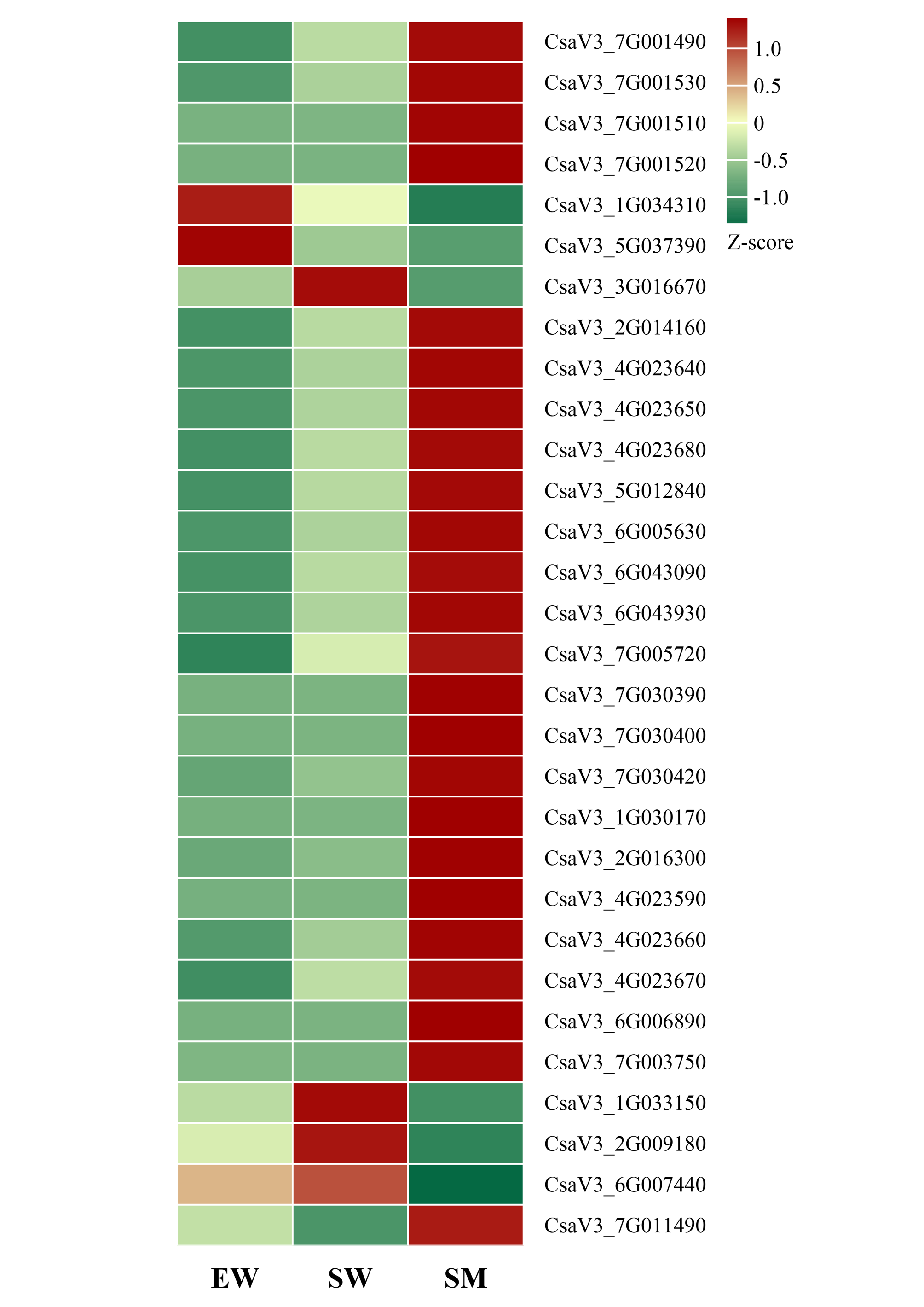

Supplement: Supplementary Figure 3 — Effects of melatonin on the expression of cell wall formation-related genes in cucumber seedings. [file Image_3.tif]
